# Electrochemical Sensing and Assessment of Parabens in Hydro-Alcoholic Solutions and Water Using a Boron-Doped Diamond Electrode

**DOI:** 10.3390/s8074330

**Published:** 2008-07-25

**Authors:** Ciprian Radovan, Dan Cinghiță, Florica Manea, Manuela Mincea, Codruța Cofan, Vasile Ostafe

**Affiliations:** 1 West University of Timişoara, Laboratory of Electrochemistry, Str. Pestalozzi nr.16, 300115 Timişoara, Romania; E-Mails: dan.cinghita@cbg.uvt.ro (D.C.); vostafe@cbg.uvt.ro (V.O); 2 “Politehnica” University of Timişoara, P-ta Victoriei, nr.2, 300006, Timişoara, Romania; E-mail: florica.manea@chim.upt.ro (F.M.); 3 University of Medicine and Pharmacy “Victor Babeş” Timişoara, P-ța E. Murgu, nr. 2, 300041 Timişoara, Romania; E-Mails: cofancodruta@umft.ro (C.C)

**Keywords:** parabens, boron-doped diamond electrode, cyclic voltammetry, chronoamperometry, water solubility, overall index

## Abstract

In this paper, the electrochemical behaviour of several parabens preservatives, i.e. esters of p-hydroxybenzoic acid, methyl-, ethyl- and propyl-4-hydroxybenzoates as methyl-, ethyl- and propyl-parabens (MB, EB, and PB), has been investigated at a commercial boron-doped diamond electrode (BDDE), especially in the anodic potential range, in both hydro-alcoholic and aqueous media. The cyclic voltammetric and chronoamperometric measurements yielded calibration plots with very good linearity (R^2^ between 0.990 and 0.998) and high sensitivity, useful for detection and analytical applications. The determination of the characteristics of individual compounds, of an “overall paraben index”, the assessment of the stability and the saturation solubility in water, and the amperometric sensing and determination in double distilled, tap and river water matrix of the relatively slightly soluble investigated parabens have been carried out using electrochemical alternative. Estimated water solubility was correlated with the octanol-water partition coefficient. Several ideas regarding stability and persistence of the presumptive eco-toxic investigated preservatives in the environment or water systems have been adjacently discussed.

## 1. Introduction

In drugs and cosmetic products processing, and modern food technology, the chemical preservation has become an important practice, starting from 1930 with addition of commonly used synthetic preservatives developed from benzoic acid and hydroxybenzoic acid as alkyl para (4)-hydroxybenzoates, so-called parabens or alkyl parabens (methyl, ethyl, etc.), and electron-donating alkyl gallates, derivatives of gallic acid, as antioxidants [1-10]. Among many additives, synthetic and naturally occurring compounds are used, especially as antioxidants [3, 6-16].

The parabens preservatives, acting simultaneously as antimicrobial agents and antioxidants, and alkyl gallates which usually accompany the parabens as typical antioxidant additives, improve the stability of the food and pharmaceutical products and prevent rancidness in product containing lipids or fats. The alkyl parabens are often used in combination. An increasing trend is seen towards the use of combination of a series of preservatives, with an extended action spectrum, because the specific activity of the parabens and the antioxidant character of these additives appear to increase with alkyl-chain length. So, the structural aspects and the lipophilicity parameters, or the difference of solubility in water, play an important concomitant role in the large action spectrum for the various lipophilic or hydrophilic characters of the products used. Metylparaben and propylparaben, for example, are often used together since they have a synergistic effect [4].

Various methods for characterization or analytical evaluation of preservatives and antioxidants have been explored and applied [1, 2, 4-6]. Alongside chromatographic or spectrophotometric alternatives, knowledge of the redox and amperometric behaviour, the electrochemical assessment can be a basis to explain useful properties or a direct analytical pathway in dosage of the additives in mixture or individual systems. Electrochemistry of various natural antioxidants is the subject of a active research as is the electrochemical study of the phenolic compounds or their derivatives [11-20]. However, there are relatively few recent papers focused on the electrochemical study of the used preservatives from the parabens series, employed in food, pharmaceutical or cosmetic products, alone or in combination with some associated antioxidant additives from the alkylgallates series [1-3, 7-10]. There is practically absent electrochemical data concerning either the direct or indirect assessment of the stability, the presence or the persistence in metabolic fluids or in water of these substances. The controlled interaction of preservatives and antioxidants with the cathodic process of electroreduction of the oxygen investigated using a mercury film electrode has been reported [1]. A glassy carbon has also been used in the electrochemical detection of parabens [2, 3].

Together with the favourable preservative and antioxidant properties of the additives, it is also important to take into account their potentially negative biological effects, and possible side effects on humans and other mammals, such as estrogenic effects which may involve breast cancer risk [21-25]. Study of such effects requires correlation with their water solubility, or lipophilicity and toxicity characteristics. In this overall context, the relationships between structure and activity, the composition and stability of the compounds, and their dosage in target applications and frequency of their use, are especially important.

In this work, the electrochemical behaviour of some preservatives-antioxidants, esters of *p*-hydroxybenzoic acid, methyl-, ethyl- and propyl-4-hydroxybenzoates (MB, EB, and PB), so-called parabens, was investigated in hydro-alcoholic and aqueous solutions, at a commercial boron-doped diamond electrode (BDDE), especially in the anodic potential range. To the best of our knowledge, no anodic sensing, characterization and determination of these commonly used preservatives-antioxidant food additives at a conductive diamond film electrode has previously been made. The principal aim of this work, which has an introductory character, was to test and collect new data for the electrochemical determination of parabens in individual and mixture systems, using as amperometric sensor a commercial BDDE designed for electroanalysis.

Formulae of the investigated compounds are: 
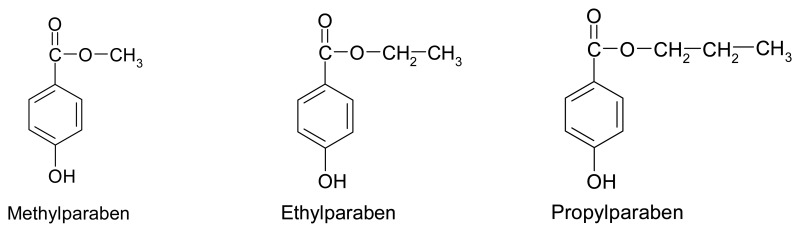


## 2. Experimental Section

The electrochemical measurements were carried out in an undivided Metrohm three electrode cell equipped with a BDDE, a 3 mm diameter stationary disc embedded in a Teflon rod as working electrode, a platinum foil counter electrode and a saturated calomel electrode (SCE) as reference. The commercial product, BDDE supplied by Windsor Scientific Ltd. for electroanalytical use was a mirror polished doped polycrystalline industrial diamond (microcrystalline) with doping degree about 0.1% boron. The working electrode, the same type as BDDE used in our previous papers [26-28], was preformed and stabilized by subjecting it to several hundred cycles between large potential limits in neutral media.

The electrochemical data resulted as cyclic voltammograms (CVs) and chronoamperograms (CAs) were obtained by using an Autolab PGstat 20 Eco Chemie device (The Netherlands) controlled by a PC running GPES 4.8 version software. The explored domains corresponded to extended potential ranges, between -1 V and +1.75 V vs. SCE, or only in more restricted anodic potential ranges, usually starting from 0 V vs. SCE in positive direction. The volume of the explored solution in the cell was large (50 mL). The chronoamperometric response was recorded for electrolysis at controlled potential, in an unstirred solution, selecting a single potential level (AUTOLAB-GPES Software-Chronoamperometry, interval time > 0.1 s) from the potential range characteristic for the current peak or the limiting current. The quantity of the analyte in the 50 mL is enough and remains practically unchanged. The method has been used also in our recent previous papers [26, 28].

Prior to the start of the electrochemical investigation and between measurements, in order to achieve very high reproducibility, the working electrode was carefully cleaned, polished with (0.3-0.1μm) alumina powder in aqueous suspension on a pad, washed with deionized water, ethanol or other organic solvents, *e.g.*, acetone, and finally with a very large volume of double distilled water.

The used substances were analytical or high purity degree Merck, Fluka and Aldrich reagents. The solutions with the exception of the stability tests described later, were normally freshly prepared using ethanol and double distilled water. The investigated target substances were esters of the parahydroxybenzoic acid, such as alkyl-para(4)-hydroxybenzoates, usually called parabens or alkylparabens, respectively methylparaben (MB), ethylparaben (EB), and propylparaben (PB), which are usually considered soluble only in alcoholic media. In addition, for the generic comparison, a number of CVs were recorded for parahydroxybenzoic acid, a characteristic compound in the parabens class and propyl gallate (PG), a representative antioxidant from gallates class, which is frequently used in combination with parabens. Ethanol and water were used as solvents for standard alcoholic or saturated aqueous stock solutions, and unbuffered 0.1 M Na_2_SO_4_ as supporting electrolyte (SE), as hydro-alcoholic solution (in 1/4 ethanol/water volume ratio, i.e. 20 % volume of ethanol in the final working solution from the cell). The use of ethanol was selected for compatibility and to prevent a separation from the solution of the target analyte and an advanced fouling of the electrode through reactants or reaction products. The working solution in the cell (50 mL) has been prepared freshly from a more concentrated Na_2_SO_4_ aqueous solution diluted adequately with water and ethanol in the above mentioned volume ratio. By adding a very small volume of the relatively concentrated samples in the large volume of the working solution from the cell, the modification in the overall ethanol content was insignificant.

The cyclic voltammetry (CV) and chroanamperometry (CA) measurements were accomplished using 50 mL volumes of solutions in the working cell and predominantly an unbuffered hydro-alcoholic neutral media described above. A 0.05 Vs^-1^ scan rate was used predominantly, but the separate scan rate effects were also investigated between 0.01 and 0.1 Vs^-1^ limits. The tests in alkaline or acidic media, only sequentially carried out when pH was changing, were performed by controlled addition of sodium hydroxide or sulfuric acid solution in 0.1 M Na_2_SO_4_ unbuffered supporting electrolyte. Working temperature was 23±1°C. The CV and CA investigations occurred predominantly at neutral pH.

## 3. Results and Discussion

### 3.1. Cyclic voltammetry and chroanoamperometry data in hydro-alcoholic solutions

**Figure 1.**
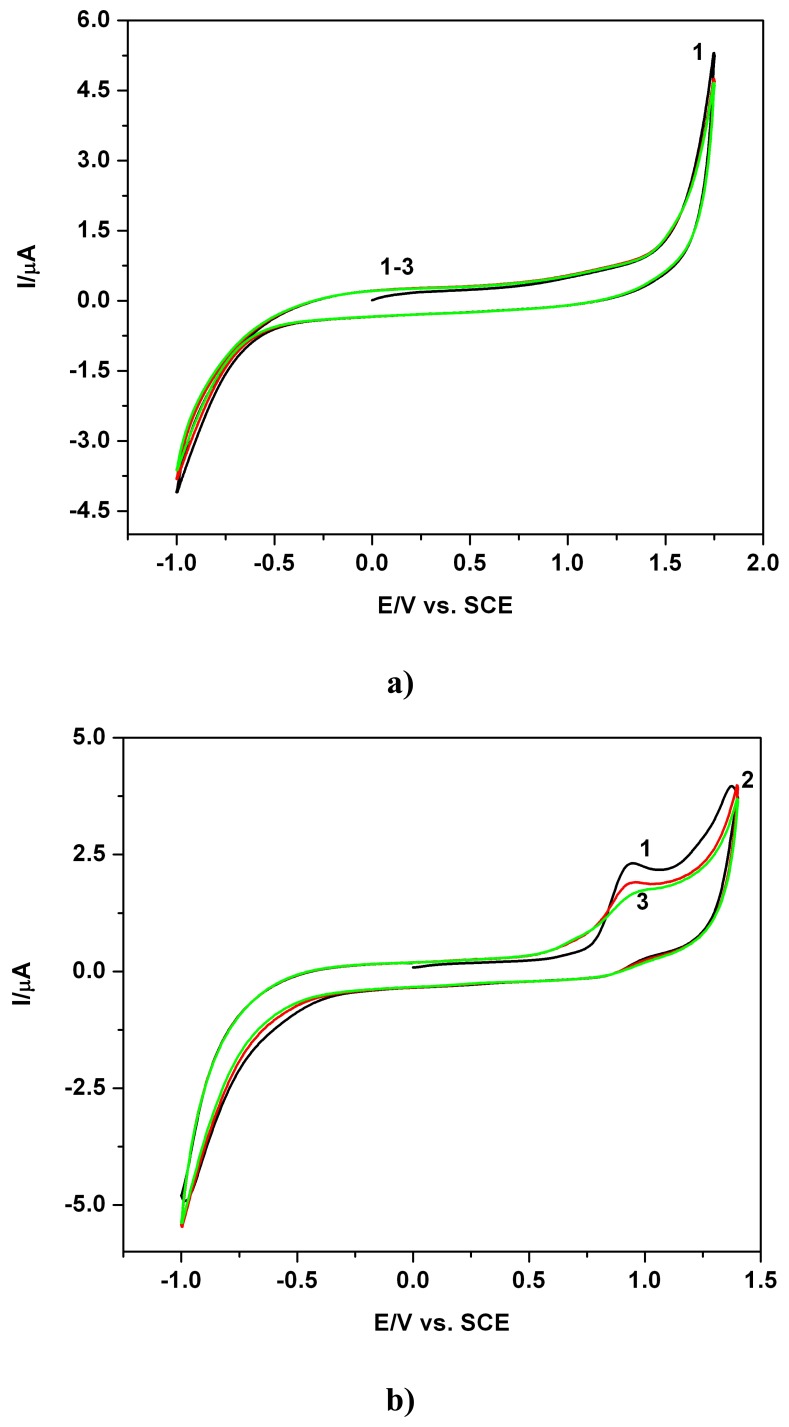

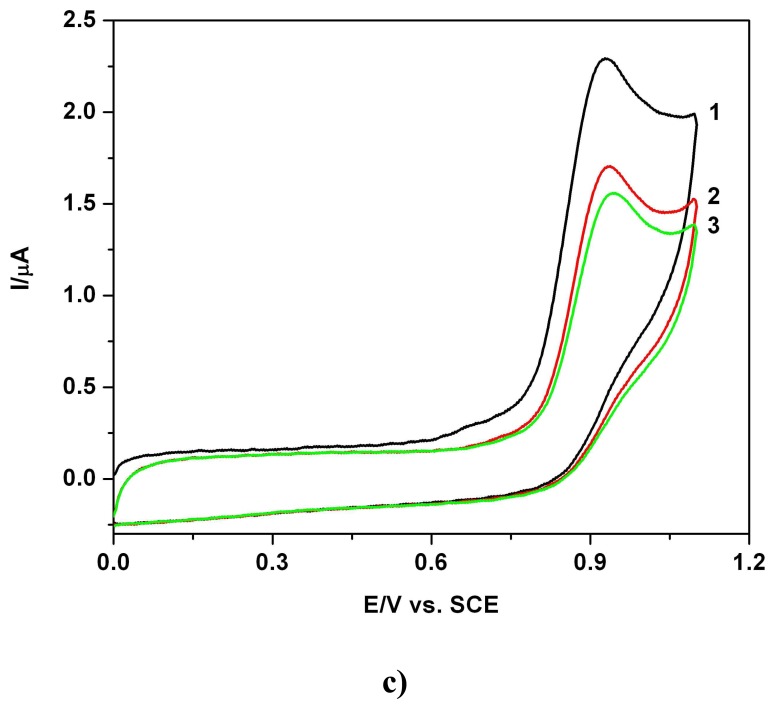
**(a)** Cyclic voltammograms (CVs): 0.1 M Na_2_SO_4_ hydro-alcoholic solution supporting electrolyte (SE-1/4 ethanol/water volume ratio, i.e. 20 % volume of ethanol in the final solution in the cell), pH 7; 1-3: scan 1-3, first scan starting from 0 V vs. SCE in positive direction, scan rate 0.05 Vs^-1^. **(b)** Cyclic voltammograms (CVs): 0.08 mM EB in 0.1 M Na_2_SO_4_ hydro-alcoholic solution supporting electrolyte, pH 7; 1-3: scan1-3, first scan starting from 0V vs. SCE in positive direction, extended potential range, scan rate 0.05 Vs^-1^. **(c)** Cyclic voltammograms (CVs): 0.08 mM EB in 0.1 M Na_2_SO_4_ hydro-alcoholic solution supporting electrolyte, pH 7; 1-3: scan 1-3 (S1, S2, S3), first scan starting from 0 V vs. SCE in positive direction, restricted potential range, scan rate 0.05 Vs^-1^.

Figure 1 depicts as examples, typical CVs involving successive first, second and third scan - curves 1 to 3, with reference to the supporting electrolyte with the accessible potential window, and the presence of EB at a relative high concentration, as curves 1-3 recorded in extended anodic and cathodic potential range (Figure1a, Figure1b) and in restricted anodic potential range (Figure 1c). All CVs were recorded starting from 0 V vs. SCE in positive direction. The anodic current peaks were delimited on the forward branches of the voltammograms and a clear irreversibility can be observed. The anodic waves decrease from first to second and third scan when a tendency of current stabilization is manifested. In the examples presented in the following figures CV data are consistently compared to the first scan.

**Figure 2.**
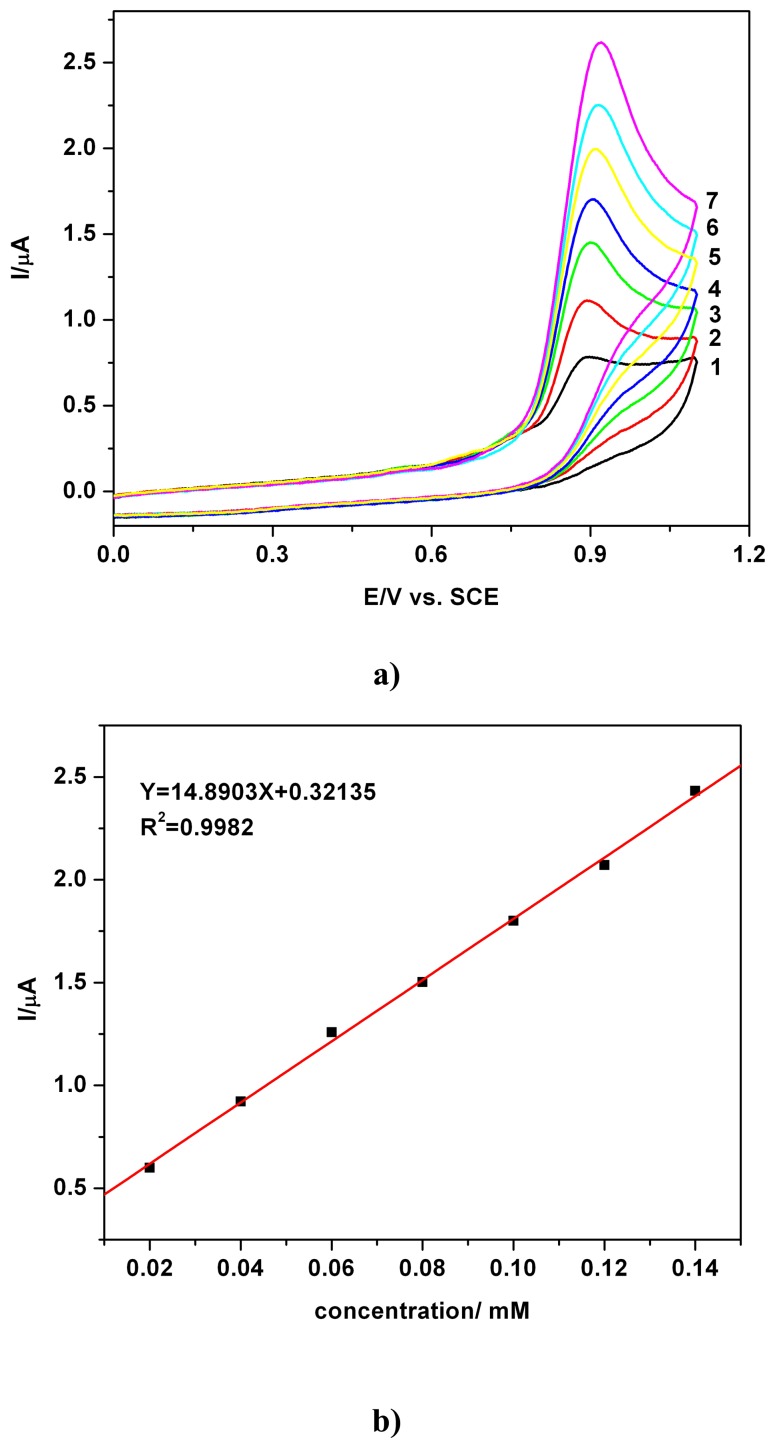
**(a)** Cyclic voltammograms (CVs): 1-0.02 mM; 2-0.04 mM, 3-0.06 mM, 4-0.08 mM, 5-0.10 mM, 6-0.12 mM, 7-0.14 mM PB in 0.1 M Na_2_SO_4_ hydro-alcoholic solution supporting electrolyte(SE-1/4 ethanol/water volume ratio, i.e. 20 % volume of ethanol in the final solution in the cell), pH 7, S1- first scan, scan rate 0.05 Vs^-1^. **(b)** Corresponding linear calibration plot of the current vs. concentration (current peak read around 0.9 V vs. SCE)

A series of resultant CVs obtained directly over the concentration range 0.02 mM - 0.14 mM for PB standard solution, corresponding to curves 1-8, is illustrated in Figure 2. Having the longer alkyl chain, PB is a representative example of the more complex parabens from the explored series. The initiation of the anodic oxidation, corresponding to an anodic wave or current peak is a known characteristic of the de-electronation and dehydrogenation step in the oxidation pathways of the oxygen containing organics [17] and phenolic compounds [3, 9-13, 15-20].

The current peaks, manifested at the potential above +0.9 V vs. SCE, increased progressively with concentration. A linear calibration plot of the current peak vs. concentration with very good correlation parameter (R^2^= 0.9982) was obtained. Similar voltammetric data and calibration plots (not represented here) were obtained for MB and EB. A summary of these results is presented in Table 1 (see below) and suggests that CV measurements can provide a very useful analytical approach. The similarity of these data with published data for MB and PB exemplified CVs at a glassy carbon electrode should be pointed out [2], but the current peaks at BDDE in our investigation were situated at less positive potentials, the lower background current, the electrode material itself, the non fouling effect and the electrolytic media certainly contributing to this shift.

**Figure 3.**
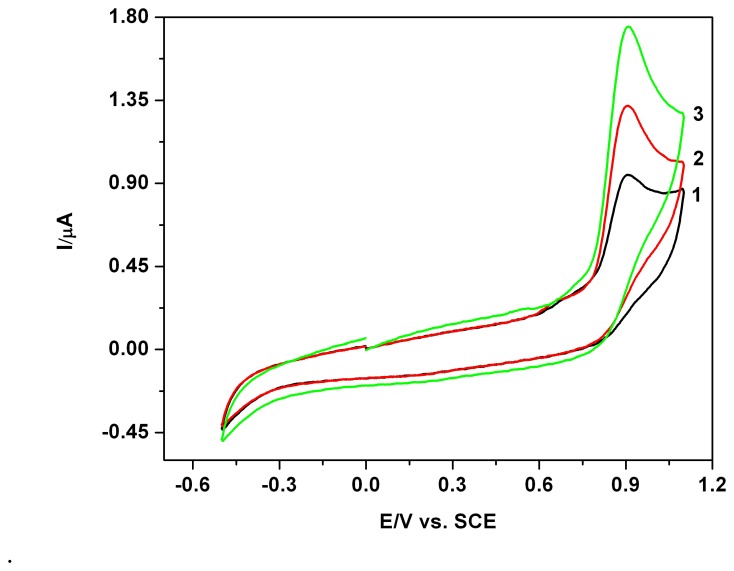
Cyclic voltammograms (CVs): parabens as mixture system in 0.1 M Na_2_SO_4_ hydro-alcoholic solution supporting electrolyte (SE-1/4 ethanol/water volume ratio, i.e.20 % volume of ethanol in the final solution in the cell), pH 7, S1, scan rate 0.05 Vs^-1^; 1-0.02 mM MB; 2-0.02 mM MB + 0.02 mM PB; 3-0.02 mM MB + 0.02 mM PB + 0.02 mM EB.

Figure 3 shows another example of CVs obtained in solutions with one, two or three parabens (curves 1-3) at comparable concentrations, which suggests an additive behaviour and group behaviour, very stimulatory for defining a non conventional global parameter which could be simply considered an “overall paraben index”. ORIGIN 7 was used for the graphical processing and analysis of the experimental data. The background current value in the potential range of the current peak was considered, by using the linear extrapolation of the first part of the forward branch of the CV (a sort of tangent line) as a simple alternative. Comparatively, the background current itself obtained previously from several CVs in supporting electrolyte and in the absence of the analyte was also considered.

The investigation of the effect of the scan rate on the current peaks was also carried out (not presented, but discussed here). The linear proportionality of the current peak vs. scan rate and square root of the scan rate indicated that the process was generally diffusion step controlled and the practically zero intercepts suggested that adsorption steps and surface interactions were negligible. Several presumptive hindering effects by insoluble reactants or reaction products and fouling were also diminished or avoided by selection of the water-ethanol mixture solvent as working medium.

**Figure 4.**
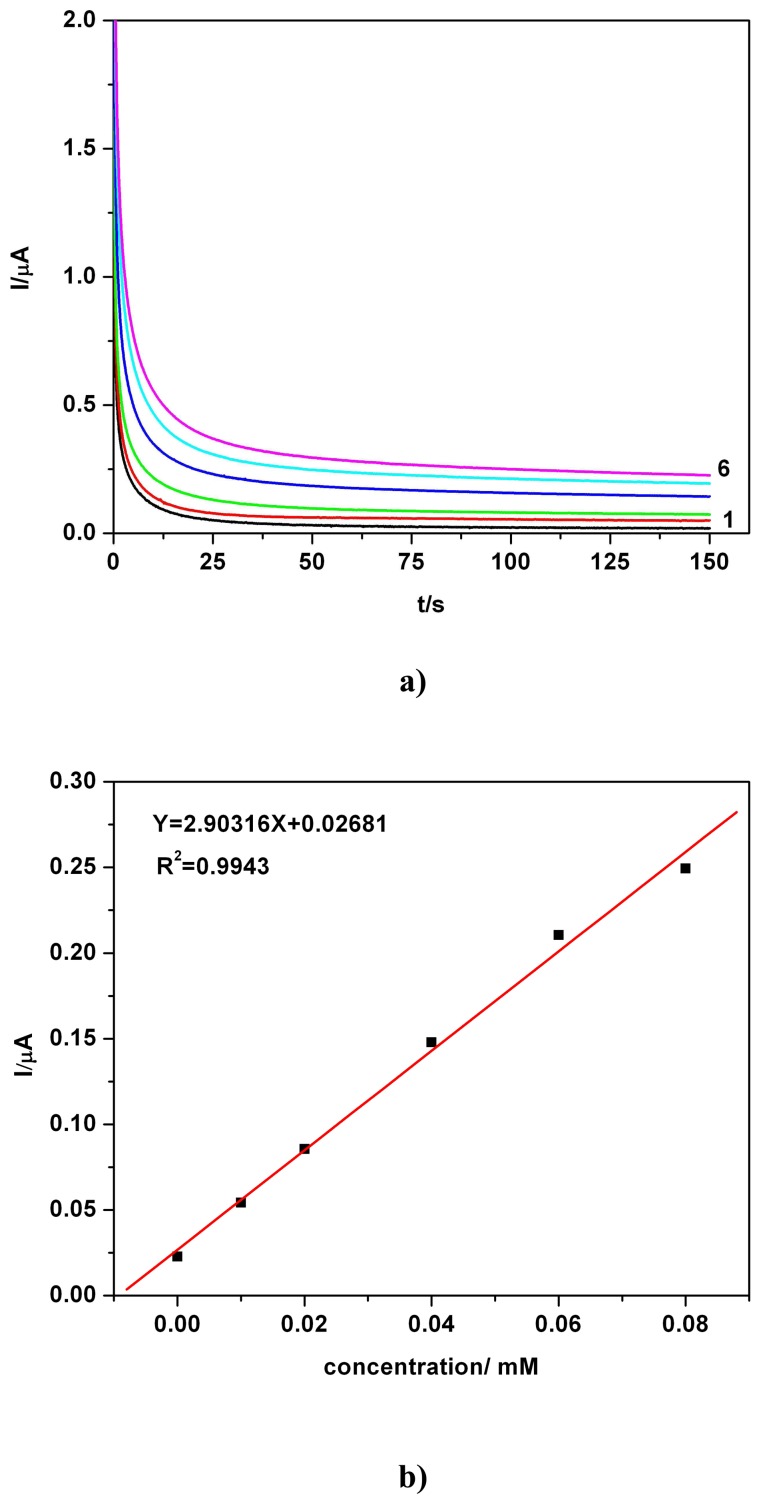
**(a)** Chronoamperograms (CAs), PB in 0.1 M Na_2_SO_4_ hydro-alcoholic solution supporting electrolyte(SE-1/4 ethanol/water *volume* ratio, *i.e.* 20 % volume of ethanol in the final solution in the cell), pH 7, at + 0.9 V vs. SCE; concentration: 1-0 mM (SE); 2-0.01 mM; 3-0.02 mM; 4-0.04 mM; 5-0.06 mM; 6-0.08 mM. **(b)** Corresponding linear calibration plot of the current vs. concentration; current read at 120s.

A first example of a series of CAs, obtained for quiescent solutions, also selected for PB in the concentration range from 0.01 mM to 0.08 mM at a potential selected from CV data is shown in Figure 4a (Precise working conditions are detailed in the figure caption). This time of readings has been selected conventionally, assuming a quasi-steady state, but several readings have been performed also for various times (data not shown). The large volume of the processed solution sustains this alternative experimentally. A similar approach was used also in our previous papers [26, 28].

The quasi-steady state is established relatively fast. The corresponding calibration plot from Figure 4b resulted from current values read at 120s. A linearity with a very good correlation parameter (R^2^=0.9943) suggested the suitable applicability of the chronoamperometry at BDDE for amperometric sensing and analytical determination.

**Figure 5.**
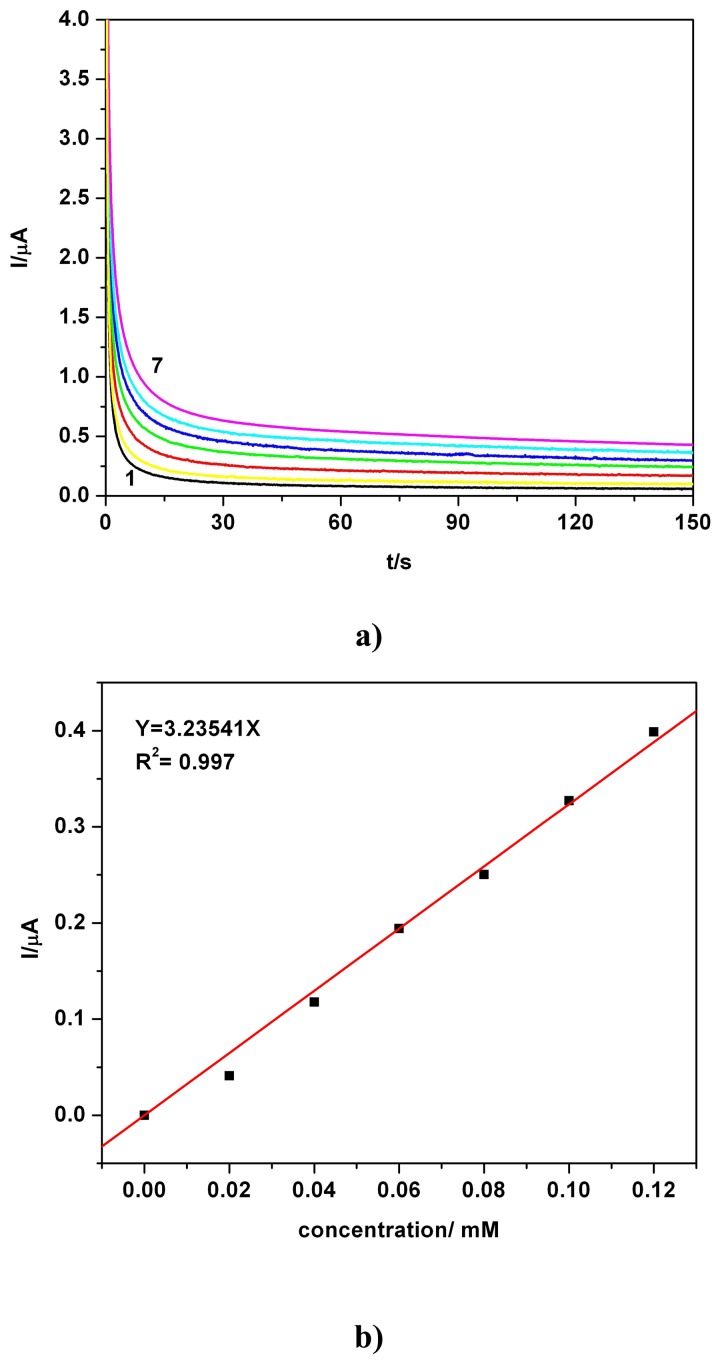
**(a)** Chronoamperograms (CAs), mixture system-MB, EB, PB in 0.1 M Na_2_SO_4_ hydro-alcoholic solution supporting electrolyte, pH 7, at 0.9 V vs. SCE ; concentration: 1-0 mM (SE), 2-0.02 mM MB, 3-0.02 mM MB + 0.02 mM EB, 4-0.02 mM MB + 0.02 mM EB + 0.02 mM PB, 5-0.02 mM MB + 0.02 mM EB + 0.04 mM PB, 6-0.02 mM MB + 0.02 mM EB + 0.06 mM PB, 7-0.02 mM MB + 0.02 mM EB + 0.08 mM PB. **(b)** Corresponding linear calibration plot of the current vs. concentration of the “overall paraben”; current read at 120s.

The approach of the additivity of the amperometric signals in mixture parabens solutions represented a further important step in exploring the CA data. Figure 5a shows a series of CAs obtained directly in progressively complicated mixture systems with various parabens contents, starting from single component to di- and tri-component system, finally defined as a complex matrix for a continuous increase of the concentration of the third component. Corresponding calibration plot of the current vs. concentration of the conventional “overall paraben”, presented in Figure 5b was linear with a high correlation parameter, R^2^= 0.997. This result recommended the CA as a suitable way for sensing and analytical assessment of soluble parabens. The following steps in our investigation were the approach of the more sensitive problems, the electrochemical assessment of the parabens in water and various aqueous systems and matrix (distilled or double distilled water, tap water, and river water) and the use of the electrochemical data for the determination of the water solubility of the parabens in the explored series.

### 3.3. Amperometric sensing in various water matrix

**Figure 6.**
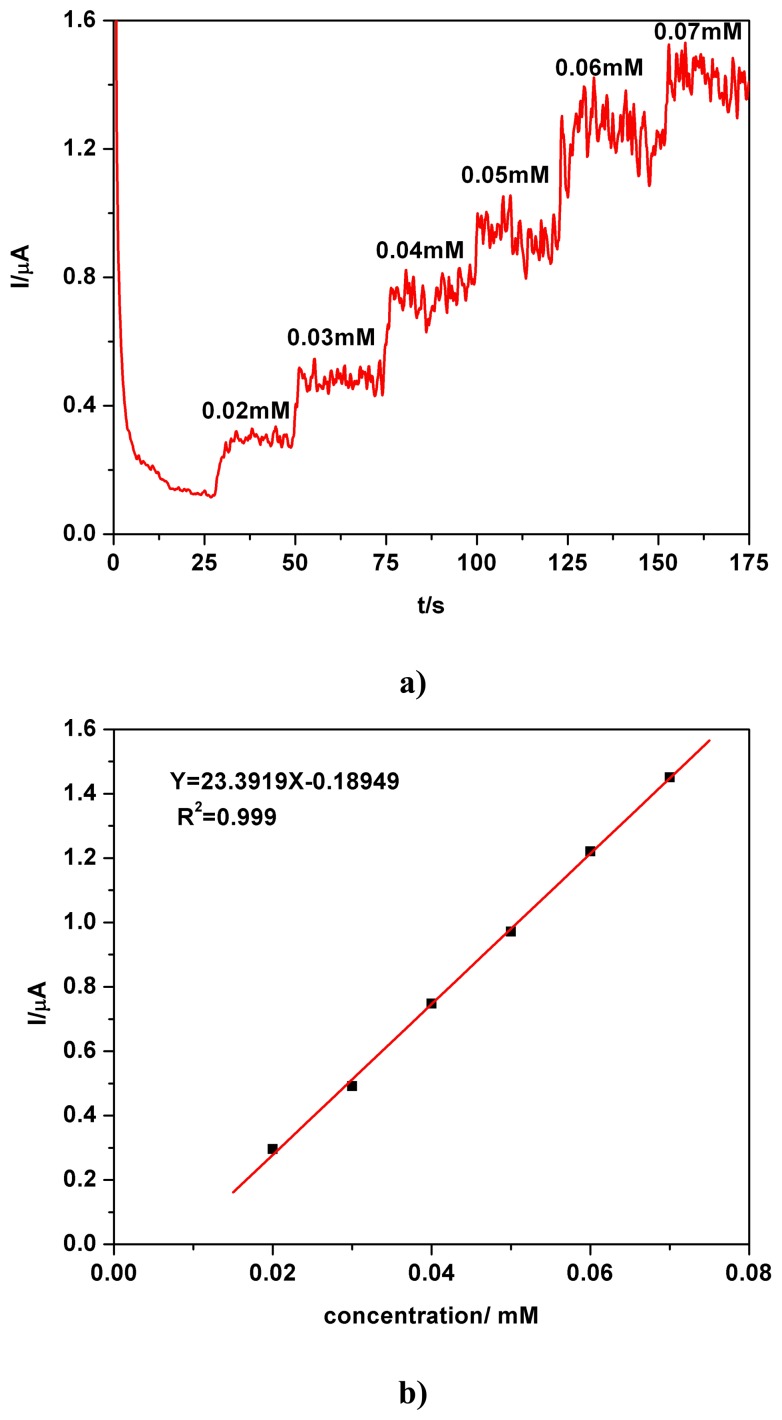
**(a)** Chronoamperogram, PB, stepwise successive addition (samples and supporting electrolyte in tap water), pH 7, magnetically stirred solution; potential +0.9 V vs. SCE. **(b)** Corresponding linear calibration plot of the current vs. concentration.

Amperometric sensing of the investigated parabens in aqueous solutions prepared from tap water and river water is shown by the examples in Figure 6a and 6b and by the CA data in Table 1. The chronoamperogram exemplified for PB, in conditions of a stepwise successive addition of stock solution samples to the supporting electrolyte, both prepared with tap water, corresponded to a progressively increase of the amperometric signal, proportional to the increased concentration. The linear calibration plot with high correlation parameter and complementary data suggest a good degree of recovery of the electroanalytical characteristics typical for chronoamperometric sensing and detection of the parabens using a BDDE and are comparable with data obtained from similar previous investigations using solutions prepared with double distilled water.

A list of equations of the linear dependencies of the current vs. concentration, and corresponding characteristic parameters, working conditions and techniques applied in various situations are summarized in Table 1. High sensitivities and very high correlation parameters were associated with the suitable electroanalytical conditions. Low limits of detection, LOD were obtained as 3σ/slope for RSD between 2-3% (from three replicates) and S/N=3 condition (The noise, N or σ, was referred to the amperometric signal which resulted at the minimum explored concentration). The higher sensitivity for CA detection in stirred solution is evidently justified by the improved hydrodynamic conditions. The matrix effect (whether in natural or tap water) played a minor role and the electroanalytical performances were practically similar to the data corresponding to solutions prepared in double distilled water. In Table 1 were presented only two examples which corresponded qualitatively to the general quantitative tendency observed in the paraben sensing by CA at a BDDE in the stirred system and tap or river waters matrix.

### 3.4. Solubility in water evaluated from electrochemical data

**Figure 7.**
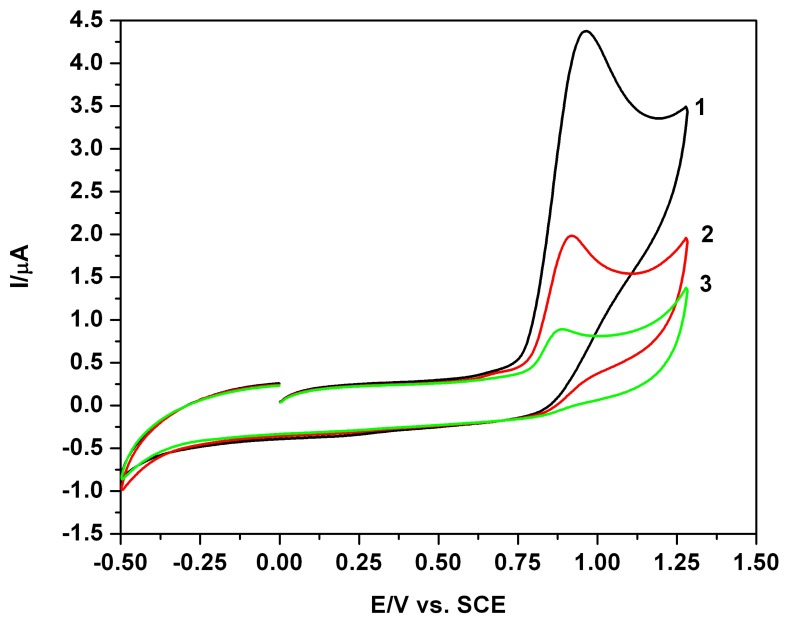
Cyclic voltammograms: 1-MB, 2-EB, 3-PB, saturated aqueous solutions (SAS) in 0.1 M Na_2_SO_4_ hydro-alcoholic solution supporting electrolyte (SE), 1mL SAS, 50 mL SE, pH 7, S1- first scan; scan rate 0.05 Vs^-1^.

Figure 7 shows an example of CVs, curves 1-3, obtained at BDDE in a similar mode to the previously discussed CVs, now recorded with saturated aqueous solutions samples resulting from MB, EB and PB dissolution, transferred by suitable dilution with supporting electrolyte directly in the electroanalytical cell. The saturated solutions of the explored compounds were collected from supernatants resulted after dissolution in stirred water and complete decantation, as liquid phase coexisting with the solid sediment. The saturation concentrations presented in Table 2 were calculated from calibration equations.

**Figure 8.**
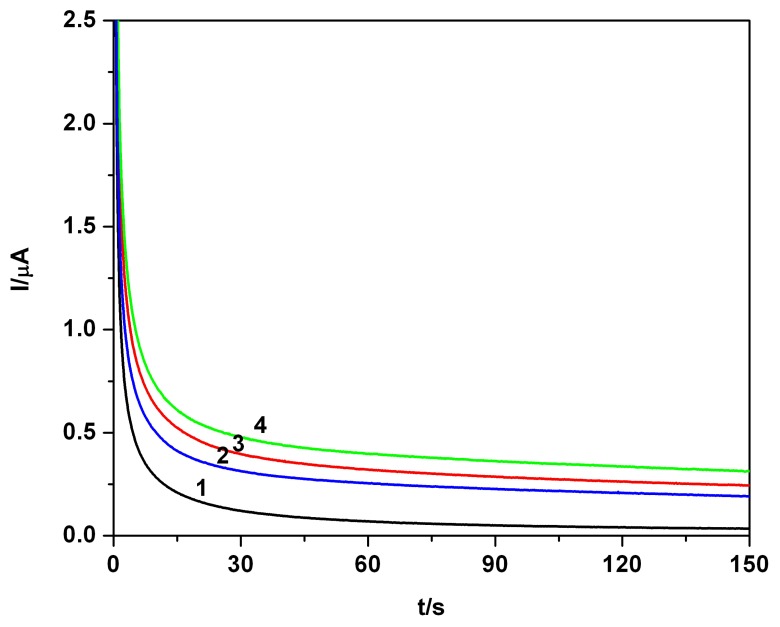
Chronoamperograms; MB SAS testing and standard addition: 1-suporting electrolyte (SE) 50 mL, 2-0.2 mL MB saturated aqueous solution (SAS) addition / 50 mL SE, 3-0.2 mL MB SAS / 50 mL SE with 0.02 mM MB standard solution content, 4-0.2 mL MB SAS / 50 mL SE with 0.04 mM MB standard solution content (current values evaluated at 120 s); +0.9 V vs. SCE electrode potential.

**Figure 9.**
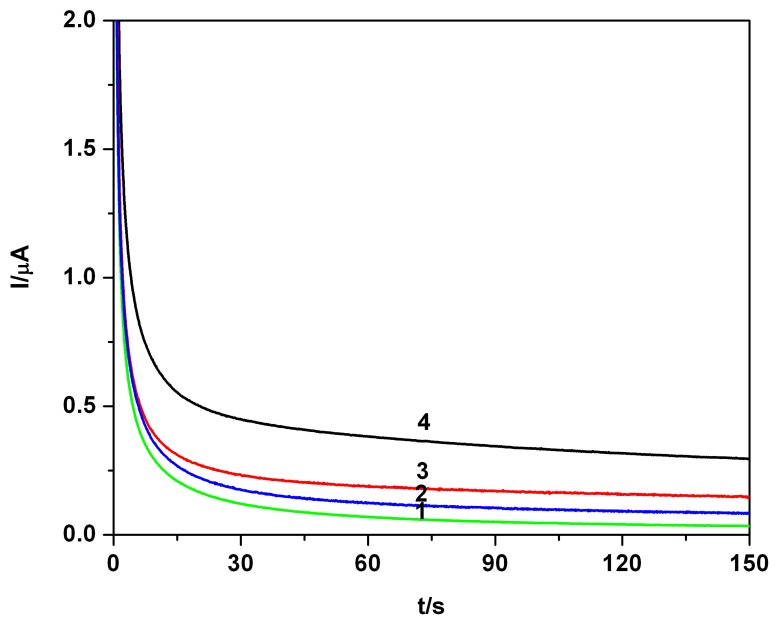
Chronoamperograms; EB SAS testing and standard addition: 1-suporting electrolyte (SE) 50 mL, 2-0.2 mL EB saturated aqueous solution (SAS) addition / 50 mL SE, 3-0.4 mL EB SAS / 50 mL SE, 4-0.4 mL EB SAS / 50 mL SE with 0.04 mM EB standard solution content (current values evaluated at 120 s); +0.9 V vs. SCE electrode potential.

A similar series of solubility data were obtained from the chronoamperograms exemplified in Figures 8 and 9 by alternate use of various samples of different parabens in the same supporting electrolyte, resulted by dilution from standard stocks and from saturated aqueous solutions, as an adequate variant of the standard addition method and comparison calculation, applied for the condition of the same selected working constant potential (from the potential range of the current peak) and the same time of the current readings. Detailed circumstances are described in the figure captions. The concentration in Table 2, regarding water solubility of the investigated parabens, resulted from CA data is close to similar values obtained from CV data. In this manner the CV and CA offer an accessible possibility for evaluation of the absent data regarding the undefined or ambiguously defined solubility of the parabens in water. On the other hand, the determined saturation concentrations were in a good accordance with calculated octanol-water partition coefficient logP, an accepted measure of the molecular hydrophobicity and a key parameter in the environmental studies of the environmental fate of chemicals [29-31]. As a general rule, logP of the compounds having hydrophobic and hydrophilic characteristics is inversely correlated to their solubility in water, a lower value of the former corresponding to a higher value of the latter. The accordance between partition coefficient and solubility resulted from data presented in Table 2 confirms the suitability of the electrochemical evaluation of the characteristics of paarbens in solution. The general feature of the parabens regarding the diminution of the solubility in water with the length of the alkyl chain is also confirmed.

It is noteworthy that solubility data of the parabens in water is practically absent from most literature dedicated to the series of the investigated alkyl-parahydroxybenzoates. Only solubility in ethanol or methanol is mentioned. In this general context, electrochemical alternative offers a consistent basis to appreciate the possibilities of obtaining, relatively easily, aqueous solutions with concentration between 1 mM (for PB, the less soluble paraben) and 10 mM (for MB, the more soluble paraben).

Besides the general aspect regarding the preliminary basis of the electrochemical sensing, the assessment of the probable presence of the parabens in aqueous media or their persistence in water systems, as a source for secondary estrogenic effects, needs to be further investigated and discussed. This last aspect regarding solubility of these slightly soluble but stable and persistent compounds in water, evaluated from electrochemically data was correlatable with hydrophilic and hydrophobic character, which can also be estimated from the good degree of accordance with the calculated partition coefficient. Higher water solubility corresponded to a lower partition coefficient.

The stability of parabens in alcoholic solutions or in water solutions (accessible data after electrochemically evaluation) was also evaluated by periodical recording of a series of cyclic voltammograms. Several alcoholic and aqueous sample solutions in the above delimited concentration ranges were prepared and their stability was tested. Sample solutions of 5 mM MB, for example, obtained by dissolving the appropriate amount of compounds in ethanol or in double distilled water remained stable during several months or a month, respectively, if stored in darkness at 4°C in a refrigerator or approximately one week for an aqueous solution if stored at 23±1°C in a covered flask. It is possible in the latter situation, for a slow hydrolysis effect in quasi-neutral aqueous solution and at a higher temperature to be observed.

The real solubility and the persistence in water systems, even as a brake in the biological treatment step of the municipal waste water treatment technology, or through accidental recycling in natural river or even in tap water, could be considered an open and important environmental aspect. These might be accessed and easily electrochemically monitored (a general aspect was also suggested by the example presented in Figures 6a and 6b).

### 3.4. Related aspects

**Figure 10.**
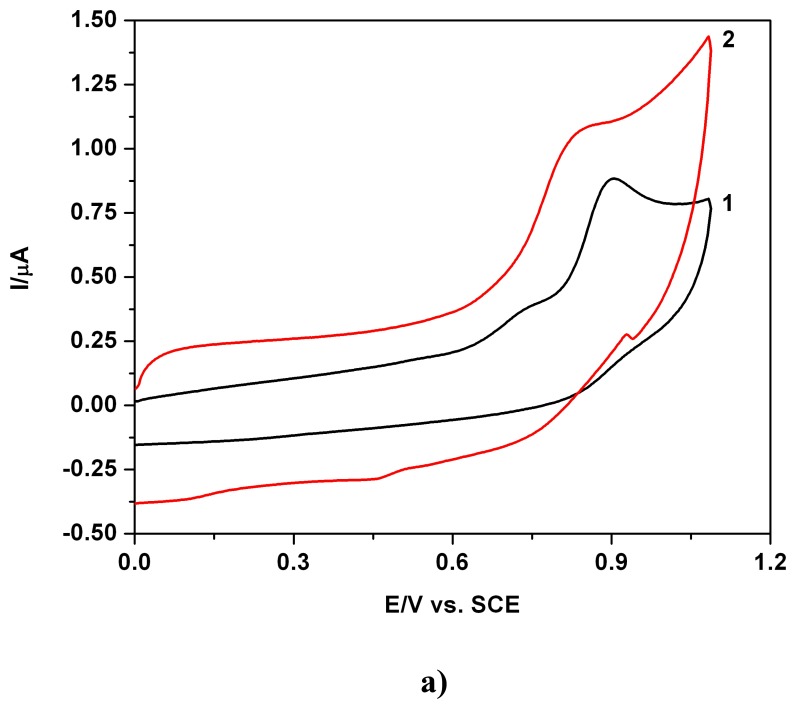

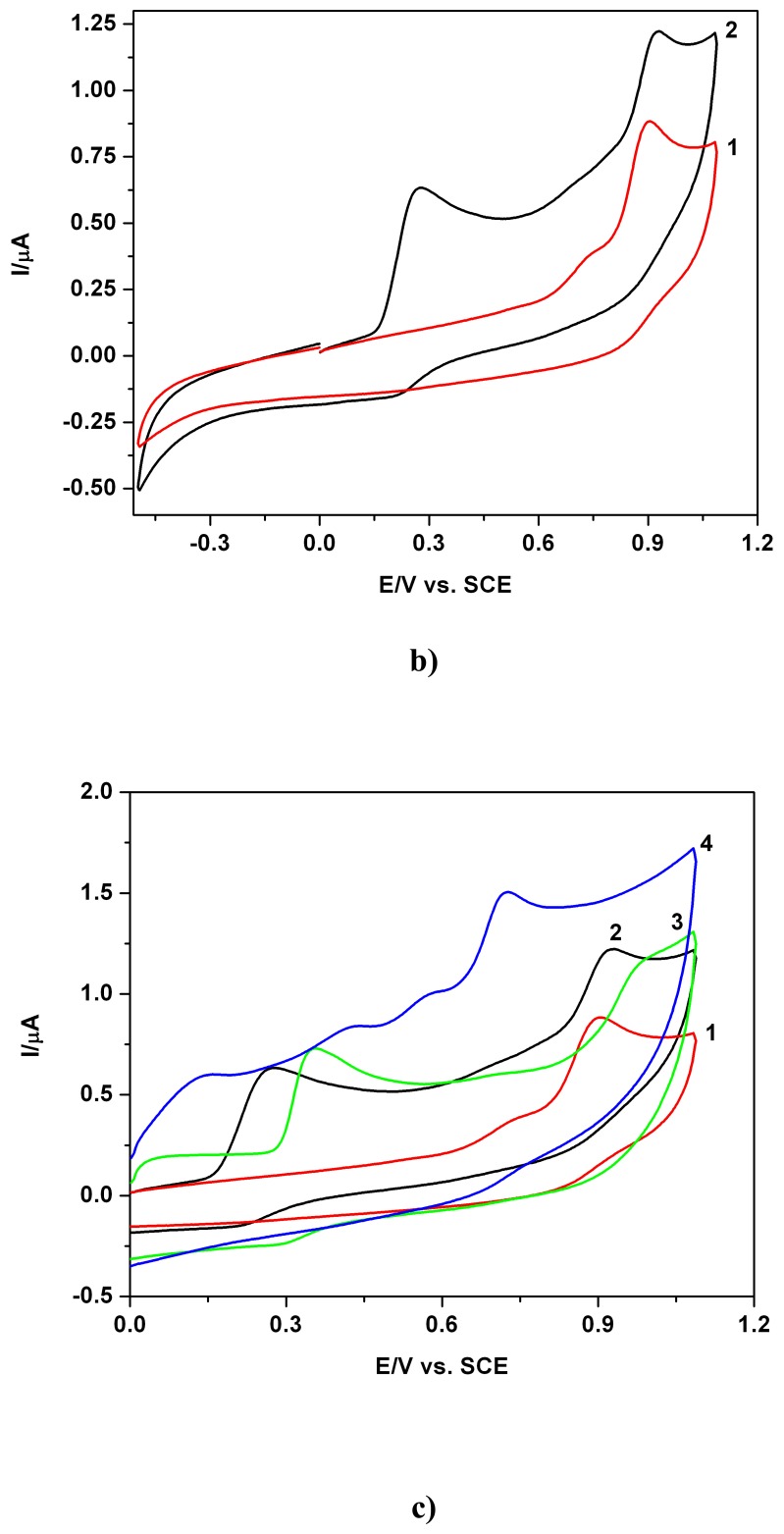
**(a)** Cyclic voltammograms: 1-0.02 mM EB; 2-Para(4)-hydroxybenzoic acid (4HBA) 0.02 mM; 0.1 M Na_2_SO_4_ hydro-alcoholic solution supporting electrolyte; S1-first scan, scan rate 0.05 Vs^-1^. **(b)** Cyclic voltammograms: 1-0.02 mM EB; 2-0.02 mM EB and 0.02 mM propylgalatte (PG); in 0.1 M Na_2_SO_4_ hydro-alcoholic solution supporting electrolyte; S1-first scan, scan rate 0.05 Vs^-1^. **(c)** Cyclic voltammograms: 1-0.02 mM EB in 0.1 M Na_2_SO_4_ hydro-alcoholic solution supporting electrolyte (HASSE), pH 7; 2-0.02 mM EB + 0.02 mM PG (pH 7); 3-0.02 mM EB + 0.02 mM PG + H_2_SO_4_ (HASSE, pH 3.82); 4-0.2 mM EB + 0.02 mM PG + NaOH (HASSE, pH 10.11); S1-first scan, scan rate 0.05 Vs^-1^.

The CVs from Figures 10a, 10b and 10c regarded as sequential typical situations, suggested certain other aspects for the further investigations. Practically, para-hydroxybenzoic acid (4HBA) offers CV similar to parabens (Figure 10a). The concomitant presence of propyl-gallate (PG), a frequently used antioxidant additive alongside parabens, is expressed by a distinct current peak to less positive potential and it can be electrochemically detected simultaneously with the accompanying paraben (Figure 10b). The pH effects in mixture system solution containing EB and PG, characterized by the modified shapes of the CVs from Figure 10c, show a shift of the current peaks in acidic media, or more complicated effects in alkaline media. In the last case the pregnant modification of the resultant CV could be supplementary associated with the hydrolysis processes of the two esters. These problems, tested only tangentially, remain further in attention and could be associated with a modification in preservative or toxic effects.

In the more complex situation, a preliminary separation of the target compounds must be realized, but the use of a BDDE provides a useful starting point for the study of the preservatives either alone or together with antioxidant compounds. This electrode could be an important component of a very stable and useful electrochemical detector.

## 4. Conclusions

Electrochemical study of the preservatives from the parabens series, the adequate selection of the supporting electrolyte or the aspects regarding the presence and concentration of the investigated compounds in the aqueous system were advantageously accomplished by the use of a commercial diamond electrode as electrochemical sensor.

The BDDE electrode is very useful for the study of the electrochemistry of parabens. The initial steps in the development of the several electroanalytical alternatives were tested. The cyclic voltammetric and chronoamperometric measurements yielded calibration plots with very good linearity and sensitivity, useful for detection and analytical applications. The assessment of the stability and the saturation solubility in both double distilled and tap water of the relatively slightly water soluble parabens investigated was easily carried out using the electrochemical alternatives. Estimated water solubility was correlated with octanol-water partition coefficient.

The group behaviour of the target compound was defined especially by CA data. An amperometric “overall paraben index” can be obtained by fast and direct measurements as voltammetric or amperometric pattern finger characteristics derived from the similar susceptibility of the various species of the series to anodic oxidation. This proposal is in accordance with recent tendencies for a global characterization of the various electrochemically approaching systems [32, 33].

Electrochemical sensing and quantitative assessment of the preservative methyl- ethyl- and propyl-benzoate from the parabens series, which are suspected as being compounds with estrogenic effects, were successfully tested in various aqueous matrix systems. The commercial BDDE offered a sustainable basis for the present and further electroanalytical development and application.

## Figures and Tables

**Table 1.** Equations of the calibration plots I = a C + b; I (μA), a (μA mM^-1^); C (mM); b (μA); sensitivity; R^2^ and LOD.

***Analyte***	***Explored concentration range (mM)***	***Regression equation of linear calibration plot***	***R****^2^*	***Sensitivity (μA***·***mM****^-1^****)***	***LOD (***μ***M)***	***Method***
MB	0.002-0.104	I = 15.211C + 0.270	0.9941	15.211	1.50	CV
EB	0.02-0.18	I = 20.645C + 0.248	0.9960	20.645	1.97	CV
PB	0.02-0.14	I = 14.890C + 0.321	0.9982	14.890	3.60	CV
MB	0.01 -0.08	I = 3.073C + 0.0185	0.9992	3.073	0.70	CA*
EB	0.002-0.112	I = 2.697C + 0.027	0.9907	2.697	1.03	CA*
PB	0.01 -0.08	I = 2.903C + 0.027	0.9943	2.903	0.97	CA*
Example (PB)	0.02 -0.07	I = 23.392C- 0.190	0.9991	23.392	-	CA**
Example (EB)	0.02-0.05	I = 13.180C+0.358	0.9977	13.180	-	CA***
MB + EB + PB ****	0.02 -0.12	I = 3.235C	0.9970	3.235	1.2	CA*

* electrode cleaning between CA recordings, quiescent solution;

** successive addition, tap water medium, intensively magnetically stirred solution;

*** successive addition, river water medium, intensively magnetically stirred solution;

**** toward an “overall paraben index”

**Table 2.** Calculated octanol - water partition coefficient, LogP* and evaluated solubility in water from electrochemical data ** C_sw_ (saturation concentration in aqueous solution) at 23±1°C.

***Compound***	O/W-LogP*	***C****_scw_****(mM)***	***Method***
MB	1.67	13.04	CV
		13.25	CA
EB	2.11	3.79	CV
		3.90	CA
PB	2.60	1.35	CV
		1.32	CA

* LogP [29-31] characteristics of hydrophobic/hydrophilic character was calculated using interactive analysis Chem Silico software-http://www.logp.com;

** solubility data were evaluated using calibration equations (from CV data) or standard addition method (from CA data)
